# Establishing a Timeline to Discontinue Routine Testing of Asymptomatic Pregnant Women for Zika Virus Infection — American Samoa, 2016–2017

**DOI:** 10.15585/mmwr.mm6611a5

**Published:** 2017-03-24

**Authors:** W. Thane Hancock, Heidi M. Soeters, Susan L. Hills, Ruth Link-Gelles, Mary E. Evans, W. Randolph Daley, Emily Piercefield, Magele Scott Anesi, Mary Aseta Mataia, Anaise M. Uso, Benjamin Sili, Aifili John Tufa, Jacqueline Solaita, Elizabeth Irvin-Barnwell, Dana Meaney-Delman, Jason Wilken, Paul Weidle, Karrie-Ann E. Toews, William Walker, Phillip M. Talboy, William K. Gallo, Nevin Krishna, Rebecca L. Laws, Megan R. Reynolds, Alaya Koneru, Carolyn V. Gould

**Affiliations:** ^1^Division of State and Local Readiness, Office of Public Health Preparedness and Response, CDC; ^2^Division of Bacterial Diseases, National Center for Immunization and Respiratory Diseases, CDC; ^3^Division of Vector-Borne Diseases, National Center for Emerging and Zoonotic Infectious Diseases, CDC; ^4^Epidemic Intelligence Service, CDC; ^5^Division of Viral Hepatitis, National Center for HIV/AIDS, Viral Hepatitis, STD, and TB Prevention, CDC; ^6^Division of HIV/AIDS Prevention, National Center for HIV/AIDS, Viral Hepatitis, STD, and TB Prevention, CDC; ^7^Division of Parasitic Diseases and Malaria, Center for Global Health, CDC; ^8^Department of Health, American Samoa Government; ^9^Pacific Islands Health Officer’s Association, Honolulu, Hawaii; ^10^Division of Toxicology and Human Health Sciences, Agency for Toxic Substances and Disease Registry; ^11^Office of the Director, National Center for Emerging and Zoonotic Infectious Diseases, CDC; ^12^Division of Tuberculosis Elimination, National Center for HIV/AIDS, Viral Hepatitis, STD, and TB Prevention, CDC; ^13^Office for State Tribal Local and Territorial Support, CDC; ^14^Division of Environmental and Occupational Disease Control, California Department of Health; ^15^Division of Congenital and Developmental Disorders, National Center on Birth Defects and Developmental Disabilities, CDC.

The first patients with laboratory-confirmed cases of Zika virus disease in American Samoa had symptom onset in January 2016 (*1*). In response, the American Samoa Department of Health (ASDoH) implemented mosquito control measures (*1*), strategies to protect pregnant women (*1*), syndromic surveillance based on electronic health record (EHR) reports (*1*), Zika virus testing of persons with one or more signs or symptoms of Zika virus disease (fever, rash, arthralgia, or conjunctivitis) (*1*–*3*), and routine testing of all asymptomatic pregnant women in accordance with CDC guidance (*2*,*3*)^.^ All collected blood and urine specimens were shipped to the Hawaii Department of Health Laboratory for Zika virus testing and to CDC for confirmatory testing. Early in the response, collection and testing of specimens from pregnant women was prioritized over the collection from symptomatic nonpregnant patients because of limited testing and shipping capacity. The weekly numbers of suspected Zika virus disease cases declined from an average of six per week in January–February 2016 to one per week in May 2016. By August, the EHR-based syndromic surveillance (*1*) indicated a return to pre-outbreak levels. The last Zika virus disease case detected by real-time, reverse transcription–polymerase chain reaction (rRT-PCR) occurred in a patient who had symptom onset on June 19, 2016. In August 2016, ASDoH requested CDC support in assessing whether local transmission had been reduced or interrupted and in proposing a timeline for discontinuation of routine testing of asymptomatic pregnant women. An end date (October 15, 2016) was determined for active mosquito-borne transmission of Zika virus and a timeline was developed for discontinuation of routine screening of asymptomatic pregnant women in American Samoa (conception after December 10, 2016, with permissive testing for asymptomatic women who conceive through April 15, 2017).

To assess whether local transmission was occurring, CDC recommended an enhanced surveillance strategy that included free clinic-based testing and reporting of all patients with an acute illness compatible with Zika virus disease at the majority of health care clinics. This enhanced surveillance was fully implemented in American Samoa by August 31, 2016. Evidence of ongoing local transmission was assessed primarily by results of rRT-PCR testing. Because of the serologic cross-reactivity between Zika virus and other flaviviruses (e.g., dengue virus) circulating in American Samoa, and the possible long duration of anti-Zika immunoglobulin M (IgM) antibody positivity, serology was considered a less specific method for detection of Zika virus and therefore a less reliable indicator of ongoing Zika virus transmission.

During August 31, 2016–October 15, 2016 (a period of 45 days, representing three 15-day extrinsic incubation periods* for Zika virus) (*4*), 32 patients were identified with symptoms of suspected Zika virus disease (one or more of the following: fever, rash, arthralgia, or conjunctivitis). Thirty (94%) of the patients were tested by rRT-PCR (median interval from symptom onset = 1 day; range = 0–30 days); two specimens did not contain sufficient quantity for testing. All 30 specimens tested negative. Among 277 asymptomatic pregnant women tested, 86 (31%) tested anti-Zika IgM-positive or equivocal; all 86 specimens subsequently tested negative by rRT-PCR. Although routine testing of asymptomatic pregnant women was not a component of enhanced surveillance for symptomatic disease, the negative rRT-PCR results were considered supportive evidence for the absence of ongoing local Zika virus transmission. Because no rRT-PCR-positive cases among pregnant or nonpregnant persons were identified within 45 days after the start of enhanced surveillance, an end date of potential active mosquito-borne transmission was calculated to be October 15, 2016 (Figure).

**FIGURE:**
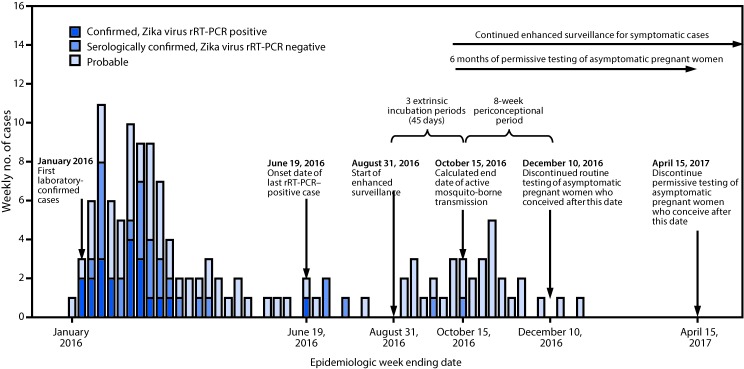
Timeline of laboratory-confirmed and probable Zika virus disease cases* with start of enhanced surveillance, calculated end date of mosquito-borne transmission, and testing recommendations — American Samoa, 2016–2017 **Abbreviation:** rRT-PCR = real-time, reverse transcription–polymerase chain reaction. * Includes reported confirmed and probable Zika virus disease cases per Council of State and Territorial Epidemiologists definitions (https://wwwn.cdc.gov/nndss/conditions/zika-virus-disease-non-congenital/case-definition/2016/06/). Laboratory criteria for confirmed Zika virus disease, noncongenital: Detection of Zika virus (ZIKV) by culture, viral antigen or viral RNA in serum, cerebrospinal fluid (CSF), tissue, or other specimen (e.g. amniotic fluid, urine, semen, or saliva); or positive ZIKV IgM antibody test of serum or CSF with positive ZIKV neutralizing antibody titers and negative neutralizing antibody titers against dengue or other flaviviruses endemic to the region where exposure occurred. Laboratory criteria for probable Zika virus disease, noncongenital: positive ZIKV IgM antibody test of serum or CSF with positive neutralizing antibody titers against ZIKV and dengue or other flaviviruses endemic to the region where exposure occurred; or negative dengue virus IgM antibody test and no neutralizing antibody testing performed.

To account for women who might have been exposed during the periconceptional period (8 weeks before conception), routine testing of asymptomatic pregnant women who conceived within 8 weeks after the calculated transmission end date (on or before December 10, 2016) was recommended (*5*). Given the possibility of Zika virus persistence in semen for 6 months (*5*), testing of asymptomatic pregnant women who conceive through April 15, 2017, can be considered as a conservative approach at the discretion of the patient and her provider (i.e., “permissive testing”).

On the basis of the information collected, testing for Zika virus infection in accordance with CDC guidance is currently recommended in American Samoa for the following groups: 1) all persons with signs and symptoms consistent with Zika virus disease; 2) asymptomatic pregnant women with an estimated date of conception (or last menstrual period) on or before December 10, 2016; 3) pregnant women with prenatal findings suggesting congenital Zika virus syndrome (*6*); 4) neonates born to mothers with laboratory evidence of Zika virus infection during pregnancy or who have abnormalities consistent with congenital Zika virus syndrome (*6*); and 5) asymptomatic pregnant women who lived in, traveled to, or had sex without a condom with a person who lived in or traveled to an area with active Zika virus transmission outside of American Samoa.

## Discussion

Because many Zika virus infections are asymptomatic (*7*), enhanced clinic-based surveillance will not detect all cases of Zika virus infection; in addition, there is always a risk of reintroduction of Zika virus to American Samoa, although a potential reintroduction is expected to be detected by continued enhanced surveillance at clinics. The calculation of an end date for local Zika virus transmission in American Samoa relied solely on rRT-PCR as evidence of recent Zika infection. Serologic testing was considered less reliable in identifying recent transmission because of the long duration of IgM positivity (12 weeks or longer) and potential for cross-reactivity with other flaviviruses. 

The increased likelihood of false positive test results that occurs as disease prevalence declines (*8*) can have negative psychosocial repercussions on pregnant women and significantly burden the health care system with only limited benefit. Establishing a timeline for discontinuing screening of asymptomatic pregnant women allows ASDoH to allocate resources appropriately toward early interventions for children and families affected by Zika virus. The surveillance processes outlined and the timeline established for American Samoa might have implications for jurisdictions where small populations and similar population immunity following widespread Zika virus exposure can facilitate interruption of transmission.

**Summary**

**What is already known about this topic?**

CDC recommends Zika virus testing of asymptomatic pregnant women who live in areas with active Zika virus transmission as part of routine obstetric care during the first and second trimesters. Currently, there are no CDC recommendations to guide the discontinuation of testing for asymptomatic pregnant women following the end of Zika virus transmission in a jurisdiction.

**What is added by this report?**

Information on Zika virus transmission from the existing enhanced surveillance in American Samoa and current CDC guidance were used to develop criteria for calculating an end date (October 15, 2016) for active mosquito-borne transmission of Zika virus and to propose a timeline for discontinuation of routine screening of asymptomatic pregnant women in American Samoa (conception after December 10, 2016, with permissive testing for asymptomatic pregnant women who conceive through April 15, 2017).

**What are the implications for public health practice?**

The rationale described in this report might be adapted by similar jurisdictions with small populations and a potential for interruption of Zika virus transmission to help guide decisions about when to discontinue routine screening of asymptomatic pregnant women for Zika virus infection following the end of active mosquito-borne transmission.

## Footnotes

* An extrinsic incubation period is the interval from acquisition of an infectious agent by a vector (e.g., mosquito) until the vector can transmit the agent to another susceptible vertebrate host.
